# Early-life dietary exposures mediate persistent shifts in the gut microbiome and visceral fat metabolism

**DOI:** 10.1152/ajpcell.00380.2021

**Published:** 2022-07-18

**Authors:** Tiffany M. Newman, Kenysha Y. J. Clear, Adam S. Wilson, David R. Soto-Pantoja, Heather M. Ochs-Balcom, Katherine L. Cook

**Affiliations:** ^1^Department of Cancer Biology, Wake Forest School of Medicine, Winston-Salem, North Carolina; ^2^Department of Surgery, Wake Forest School of Medicine, Winston-Salem, North Carolina; ^3^Comprehensive Cancer Center, Wake Forest School of Medicine, Winston-Salem, North Carolina; ^4^Department of Epidemiology and Environmental Health, School of Public Health and Health Professions, University at Buffalo, Buffalo, New York

**Keywords:** inflammation, in utero exposure, maternal diet, microbiome, obesity

## Abstract

In utero dietary exposures are linked to the development of metabolic syndrome in adult offspring. These dietary exposures can potentially impact gut microbial composition and offspring metabolic health. Female BALB/c mice were administered a lard, lard + flaxseed oil, high sugar, or control diet 4 wk before mating, throughout mating, pregnancy, and lactation. Female offspring were offered low-fat control diet at weaning. Fecal 16S sequencing was performed. Untargeted metabolomics was performed on visceral adipose tissue (VAT) of adult female offspring. Immunohistochemistry was used to determine adipocyte size, VAT collagen deposition, and macrophage content. Hippurate was administered via weekly intraperitoneal injections to low-fat and high-fat diet-fed female mice and VAT fibrosis and collagen 1A (COL1A) were assessed by immunohistochemistry. Lard diet exposure was associated with elevated body and VAT weight and dysregulated glucose metabolism. Lard + flaxseed oil attenuated these effects. Lard diet exposures were associated with increased adipocyte diameter and VAT macrophage count. Lard + flaxseed oil reduced adipocyte diameter and fibrosis compared with the lard diet. Hippurate-associated bacteria were influenced by lard versus lard + flax exposures that persisted to adulthood. VAT hippurate was increased in lard + flaxseed oil compared with lard diet. Hippurate supplementation mitigated VAT fibrosis pathology. Maternal high-fat lard diet consumption resulted in long-term metabolic and gut microbiome programming in offspring, impacting VAT inflammation and fibrosis, and was associated with reduced VAT hippurate content. These traits were not observed in maternal high-fat lard + flaxseed oil diet-exposed offspring. Hippurate supplementation reduced VAT fibrosis. These data suggest that detrimental effects of early-life high-fat lard diet exposure can be attenuated by dietary omega-3 polyunsaturated fatty acid supplementation.

## Graphical abstract

**Figure:**
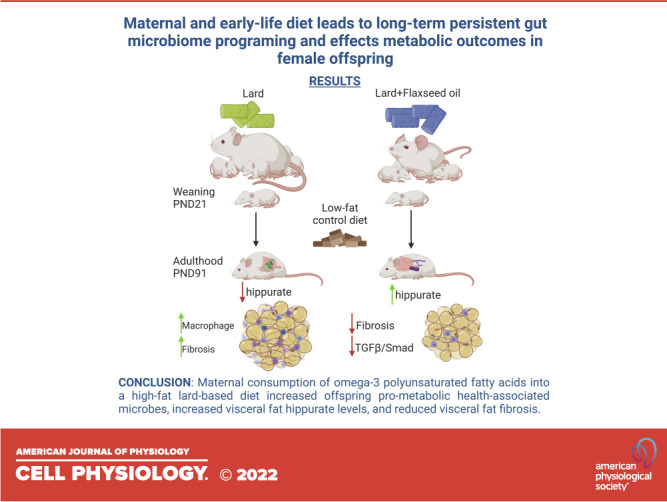

## INTRODUCTION

The Developmental Origins of Health and Disease Theory states that the maternal intrauterine environment influences fetal development into adulthood, affecting metabolic disease risk (1). A key component of the maternal intrauterine environment is the nutrition that is provided to the growing fetus through the placenta. Both maternal over- and undernutrition as well as a macronutrient imbalance can have adverse effects on the offspring.

Maternal obesity is linked to fetal overgrowth, a condition that increases the offspring’s likelihood of developing obesity later in life (2). Maternal obesity correlates with hyperphagia, increased adiposity, insulin resistance, and hypertension in adult offspring (3). In the absence of obesity, maternal high-fat diet has been linked to the development of the metabolic syndrome in offspring later in life, correlating with increased liver mass and triglyceride content, insulin resistance, increased visceral fat mass, hepatic steatosis, and adipocyte hypertrophy (3).

The detrimental effects of maternal diet are not limited to fat content. A gestational high-sugar diet containing simple sugars (dextrose and maltodextrin) altered the epigenetic modification of lipogenic and adipogenic genes in the white adipose tissue of adult rats, leading to an increased prevalence of obesity in males (4). A gestational diet containing 50% fructose produced hyperglycemia in male and female adult offspring (5). The standard “Western Diet” is high in refined sugars and saturated fats. When a high-fat, high-sucrose diet is used as a model for maternal diets, a higher prevalence of type II diabetes, obesity, and metabolic syndrome was observed in the adult offspring of mice, rats, and humans (1, 6–11).

The gut microbiome composition varies in the presence of obesity (12). Furthermore, the composition of the gut microbiota regulates the ability to harvest energy from food and the amount of energy stored as fat within the host (12). During the first year of life, breastfeeding constitutes a major portion of the infant gut microbiome (13). A review of 44 studies with a total of 2,655 human mothers identified the main bacterial genera in human breast milk as *Staphylococcus, Lactobacillus, Streptococcus, Pseudomonas, Corynebacterium, Bifidobacterium, Enterococcus, Rothia, Acinobacter, Cutibacterium, Veillonella*, and *Bacteroides* (14). A study of 107 U.S. mothers demonstrated that the dominant phylum in breast milk was Proteobacteria (Enterobacteriaceaea and Pseudomonaceae), whereas the areolar skin primarily consisted of Firmicutes (Staphylococcaceae and Streptococcaceae) (15). This study concluded that the breast milk microbial population contributed a mean of 27.7% to the infant gut microbiome, whereas the areolar skin contributed 10.3% (15). Evidence suggests that the bacterial composition of breast milk may vary according to the dietary intake and BMI of the mother but currently available studies are limited and contradictory findings were reported (14). The shift to a microbiome resembling an adult occurs with the cessation of breastfeeding rather than the introduction of alternative foods (13). Breastmilk serves not only as a source of microbial diversity but also as a means of modulation of the gut microbiome by providing the infant with lactoferrin, lysozyme, milk glycans, and soluble immunoglobulins (16). Certain families of microbes such as the *Lachnospiraceae* family are inflammatory and adipogenic (17). Species of *Lachnospiraceae* and a proportional elevated abundance of the Firmicute phyla are elevated in overweight or obese children born to overweight or obese mothers (17). Collectively, this evidence supports the hypothesis that the overweight or obese phenotype can be transmitted to offspring via modulation of gut microbiome diversity in utero and postnatally via breastfeeding.

To determine whether in utero and early-life (up to weaning) maternal diets have long-term impacts on offspring gut microbiome, adiposity, and metabolism, we placed dams on a low-fat, low-sugar control diet (control), a high sucrose, low-fat diet (HS), a high-fat lard-based diet [Lard obesity-inducing diet (OID)], and a high-fat lard + flaxseed oil diet (Flaxseed OID) beginning 1 mo before pregnancy. Dams were on the experimental diets throughout pregnancy, birth, and up to offspring weaning at *postnatal day 21*. Offspring were then placed on a low-fat, low-sugar control diet until adulthood (13 wk of age, 10 wk of control diet administration).

Our current study demonstrates that early-life exposure to a high-fat lard diet results in persistent microbiota shifts present even after 10 wk of control diet feeding that were not observed in offspring of lard + flaxseed oil OID-fed dams. Moreover, untargeted metabolomics of visceral fat from adult mice that were exposed in utero to a lard + flaxseed oil OID highlighted increased bacterially processed metabolites such as hippurate. Treatment of low-fat and high-fat diet-consuming mice with hippurate reduced visceral adipose tissue (VAT) profibrotic histopathology, suggesting a critical interaction between dietary-programmed microbiota-processed metabolites and extracellular matrix protein signaling. Taken together, we show that maternal high-fat lard diet consumption results in long-term gut microbiome programming in offspring that impacts metabolic function and inflammation that can be prevented by omega-3 polyunsaturated fatty acid supplementation.

## EXPERIMENTAL PROCEDURES

### Materials

Hippuric acid was purchased from Sigma Aldrich (Cat. No. 112003). Antibodies were obtained for CD68 (Abcam; ab31630), phospho-SMAD2 (Cell Signaling Technologies; Cat. No. 3108), SMAD2 (Cell Signaling Technologies; Cat. No. 3103), transforming growth factor-β (TGF-β) (Cell Signaling Technologies; Cat. No. 3711), and collagen 1A (COL1A) (Cell Signaling Technologies; Cat. No. 91144). Harris Hematoxylin was purchased from Newcomer Supply (Cat. No. 1201). Xylene was purchased from Fisher Scientific (Cat. No. 05082-4). Eosin-Phloxine Working Solution was obtained from Newcomer Supply (Cat. No. 1082). Glacial acetic acid was purchased from Newcomer Supply (Cat. No. 10010 A). Sirius Red F3B (Cat. No. 36-554-8), a saturated aqueous solution of picric acid (Cat. No. P6744), and solid picric acid (Cat. No. 239801) were purchased from Sigma. Histoclear was obtained from National Diagnostics (Cat. No. HS-200). The DAB Staining kit (Ref: K4065), Protein Block reagent (Ref: X0909), and Antibody Diluent (Ref: 80809) were purchased from Dako. Cytoseal XYL Mounting Media was purchased from Thermo Scientific (Ref: 8312-4).

### Animals

Female 8-wk-old BALB/c mice were purchased from Jackson Laboratories. All Teklad custom diets were purchased from Envigo. Female mice were placed on a control diet (10% kcal from fat, TD.08806), a high sugar (TD.160065), lard obesity-inducing diet (Lard OID, 60% kcal from fat, TD.06414), or a Lard + Flaxseed oil diet (Flaxseed OID, 60% kcal from fat, TD.160066) ad libitum. At 12 wk of age, mice were paired with males (consuming a low-fat control diet) for mating. Pregnancy was confirmed 1-wk post pairing and males were removed from cages. Dams were maintained on their respective diets throughout pregnancy and lactation until weaning. Pups were counted and weighed on *postnatal day 4* (*PND4*). Female pups were weaned at *postnatal day 21* (*PND21*) and were administered a control diet.

**Figure 1.**
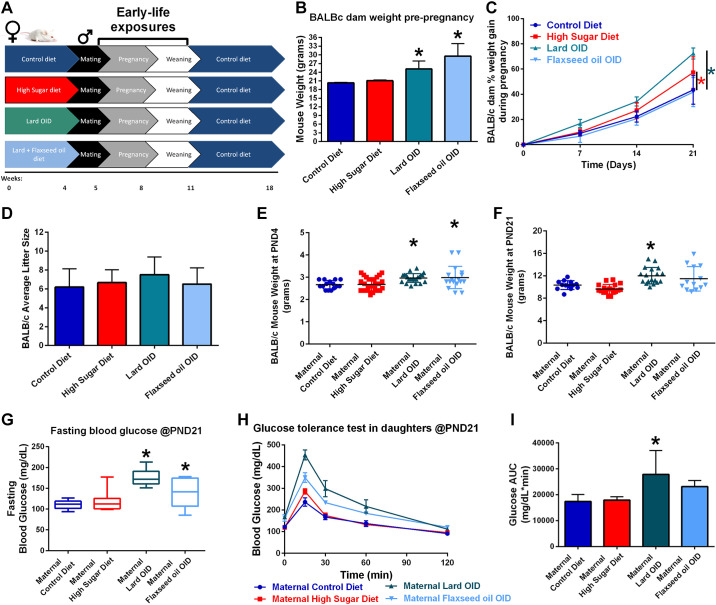
Maternal diet nutrient composition impacts metabolism of both mother and female offspring. *A*: BALB/c mouse model of in utero and early-life dietary exposures. *B*: body weight of adult female BALB/c mice before mating. *C*: percentage of weight gain of BALB/c mice during pregnancy based on measurements taken every 7 days. *D*: average number of pups born to BALB/c mice. *E*: body weight of female BALB/c offspring measured at 4 days of age. *F*: body weight of female BALB/c pups measured at *postnatal day 21* (*PND21*) days of age. *G*: blood glucose concentration of 21-day-old female offspring following overnight fasting. *H*: blood glucose concentration of female offspring over 2 h following intraperitoneal injection of 2 mg/kg sucrose solution. *I*: comparison of glucose tolerance test curves by calculation of the area under each curve. *P < 0.05. OID, obesity-inducing diet.

At weaning, pup body weights were recorded and glucose tolerance tests were administered. Glucose tolerance testing consisted of overnight fasting followed by measurement of fasting blood glucose in blood collected via tail snip and measured using a OneTouch Ultra2 (LifeScan, Inc. with GenUltimate! Test Strips; Cat. No. 100-10). An intraperitoneal injection of 2 g/kg sucrose solution was administered, and blood glucose measurements were repeated at 15-, 30-, 60-, and 120-min postinjection for comparison via blood glucose curves. Body weight was recorded weekly. At *postnatal day 91* (*PND91*) or 13 wk of age, repeat body weight measurements and glucose tolerance tests were performed. Following the procedure, mice were humanely euthanized, and their livers, VAT, and mammary glands were harvested, weighed, and preserved for future analysis. See Fig. 1*A* for the study schematic. The protocol was approved by the Animal Care and Use Committee of the Wake Forest School of Medicine and all procedures were carried out in accordance with relevant guidelines and regulations.

### Fecal 16S Sequencing

Fecal samples were collected from pregnant BALB/c dams on *day 10* of pregnancy and from female offspring at weaning (*PND21*; 3 wk of age) and adulthood (*PND91*; 13 wk of age) in a sterile hood. At collection, samples were stored in sterile cryovials and immediately placed on dry ice. Samples were stored at −80°C until they were submitted for 16S sequencing. Mouse fecal 16S sequencing was performed by Microbiome Insights Inc. (Vancouver, BC, Canada). In brief, DNA was isolated from the feces using the MoBio Powersoil extraction kit. 16S rRNA genes were PCR- amplified with dual-barcoded primers targeting the V4 region, as previously described (18–20). Amplicons were sequenced with an Illumina MiSeq using the 250-bp paired-end kit (v. 2). Bacterial sequences were denoised, taxonomically classified using Greengenes (v. 13_8), and clustered into similar operational taxonomic units (OTUs) with the mother software package (v. 1.39.5) following the recommended protocol (https://www.mothur.org/wiki/MiSeq_SOP). The resulting data set had 10564 OTUs (including those occurring once with a count of 1, or singletons). An average of 22,733 quality-filtered reads were generated per sample. The potential for contamination was addressed by cosequencing DNA amplified from specimens and from each of four template-free controls and extraction kit reagents processed the same way as the specimens. Two positive controls, consisting of cloned SUP05 DNA, were also included (number of copies = 2 × 10^6^). Operational taxonomic units were considered putative contaminants (and were removed) if their mean abundance in controls reached or exceeded 25% of their mean abundance in specimens.

### Untargeted Metabolomics

Snap-frozen VAT from adult daughters was subjected to untargeted metabolomics (Metabolon, Raleigh, NC). In brief, a Waters ACQUITY ultra-performance liquid chromatography (UPLC) system and a Thermo Scientific Q-Exactive mass spectrometer interfaced with a heated electrospray ionization (HESI-II) source and Orbitrap mass analyzer were used for analysis. Compounds were identified by comparison with library entries of purified standards and peaks were quantified by measuring the area under the curve. The informatics system consisted of the Laboratory Information Management System (LIMS), the data extraction and peak-identification software, data processing tools for QC and compound identification, and a collection of information interpretation. Log transformation and imputation of missing values were performed with the minimum observed value for each compound. One-way ANOVA with Tukey’s multiple-comparisons post hoc analysis was used to identify biochemicals that differed significantly between visceral adipose tissue samples from adult female offspring with different maternal diet exposures.

### Immunohistochemistry

VAT was harvested following euthanasia of the BALB/c offspring. Half of the tissue was fixed in 4% paraformaldehyde for 24 h and then moved to 70% ethanol. Fixed tissue was paraffin-embedded and 5 µm slices were placed on slides (2 tissue segments per slide). This process was repeated for five animals per group for a total of 40 tissue segments.

### Hematoxylin and Eosin Staining

VAT segments were stained with hematoxylin and eosin to visualize adipocyte size. Five images of each slide were taken at ×20 magnification. ImageJ software was used to measure the diameter at the widest point on 20 representative adipocytes per image. Adipocyte diameters were compared using two-way ANOVA (main column effect) with a Holm–Šídák’s multiple comparisons (GraphPad Prism Software). The average adipocyte diameter of each group (daughters of mothers receiving control, high sugar, lard, and lard + flaxseed oil diets) was compared with every other group.

### Picrosirius Red Collagen Staining

Picrosirius red collagen staining was performed on VAT sections, as previously described (21). Five images of VAT were taken per slide at ×10 magnification (5 images per mouse, 5 mice per group). InForm analysis software, v. 2.4.6781.17769 was used to process images. A threshold of optical density = 0.196 was designated as the minimum positive value and the percent of positive pixels per image was calculated. Two-way ANOVA (main column effect) with a Holm–Šídák’s multiple comparisons was used to examine differences in percent positive staining according to diet exposure group. GraphPad Prism software was used to visualize results.

### CD68 DAB Macrophage Staining

DAB staining was performed following the manufacturer’s instructions. An additional step, the Protein Block solution, was added and the slides were incubated for 5 min following the Endogenous Enzyme Block. Anti-mouse CD68 antibody was diluted in Dako Antibody Diluent (1:100) and applied to slides (24-h incubation at 4°C). Slides were exposed to DAB+ chromagen substrate solution for 9 min. Five images were taken per slide at ×20 magnification (1 slide per animal, 5 animals per group). Images were processed using InForm analysis software to generate composites. CD68+ cells were counted manually in each image. Adipocytes were counted and CD68 positive cells were normalized to total adipocytes in each image (CD68+ cells/adipocytes). Two-way ANOVA (main column effect) with a Holm–Šídák’s multiple-comparisons post hoc test was performed to compare CD68 positivity between diet exposure groups. Graphs were generated using GraphPad Prism software.

### In Vivo Hippurate Model

Low-fat or high-fat diet-fed female BALB/c mice at 12 wk of age were randomized into saline or hippurate treatment groups (*n* = 3 or 4 mice/group). At 14 wk of age, mice received a series of three intraperitoneal injections of either 100 µL saline (control) or 100 µL 10 mM hippurate at 0, 24, and 48 h. At 72 h, mice were humanely euthanized and VAT was fixed in 4% paraformaldehyde for 24 h and then moved to 70% ethanol. Fixed tissue was paraffin-embedded and 5 µm slices were placed on slides. Tissue sections were stained for fibrosis (Picrosirius red; see protocol above) or for collagen 1A (Cell Signaling, Cat. No. 91144, dilution: 1:100) using a DAB-chromogen staining protocol following the manufacturer’s instructions. Snap-frozen VAT was collected for protein analysis to measure the expression of transforming growth factor-β, phosphorylated SMAD2/3, SMAD 2/3, and collagen 1A using Western blot analysis.

### Ex Vivo Hippurate Model

Control diet-fed female 12-wk-old BALB/c mice (*n* = 3) were humanely euthanized and VAT was harvested for ex vivo experimentation. Tissue was sectioned into 10 mg segments and 4–5 segments were placed in each well of a 6-well tissue culture plate containing 3 mL RPMI media per well. VAT from each animal was left untreated (control), treated with 10 µM hippurate, or treated with 100 µM hippurate. The plate was incubated overnight at 37°C with 5% CO_2._ Protein was harvested the following morning in RIPA buffer with protease inhibitors. Western blotting was performed for quantification of pSmad2, TGF-β, and COL1A.

### Western Blot Hybridization

VAT from the in vivo and ex vivo hippurate models was sonicated in RIPA buffer to homogenize tissue. Protein was size fractionated by gel electrophoresis and transferred to a nitrocellulose membrane. Membranes were placed in blotto for 30 min to block, and then transferred to primary antibody solution for overnight incubation at 4°C (phospho-SMAD2, SMAD2, TGF-β, and COL1A from Cell Signaling Technology, dilution: 1:1,000). Membranes were washed and incubated with polyclonal horseradish peroxidase-conjugated secondary antibodies. Immunoreactive products were visualized by chemiluminescence with SuperSignal Femto and were quantified using the Bio-Rad digital densitometry software. Western blots are shown in figures as cropped images.

## RESULTS

### Maternal Diet Nutrient Composition Impacts the Metabolic Health of BALB/c Dams and Female Offspring

Female BALB/c mice were randomized into diet groups (control, HS, Lard OID, or Flaxseed OID) at 8 wk of age. After 4 wk of diet administration, prepregnancy BALB/c body weights were recorded (Fig. 1*B*). Dams receiving control diet had a mean body weight of ∼20 g; both Lard OID (25 g) and Flaxseed OID (30 g) were higher by comparison (*P* < 0.05). Following mating, dam body weight was monitored at weekly intervals, and % weight gain during pregnancy was reported (Fig. 1*C*). Dams consuming either a Lard OID (70%) or HS diet (55%) had a greater percentage of weight gain throughout pregnancy than control diet mice (45%, *P* < 0.05). Mice receiving Flaxseed OID did not significantly vary from the control group in pregnancy weight gain.

To reduce postpartum stress, dams were given four undisturbed days with pups before initial observations. At *postnatal day 4* (*PND4*), pups were counted (Fig. 1*D*). Dams in all groups birthed an average of 6–8 pups; no significant difference in litter size was observed. Pup body weight was recorded at *PND4* (Fig. 1*E*). Pups born to dams consuming either Lard OID or Flaxseed OID weighed an average of 0.3 g more than pups birthed by control-fed dams (*P* < 0.05).

At *PND21*, female offspring born to lard OID-fed mothers were observed to have an average body weight 2 g higher than that of daughters of control diet-fed mice (*P* < 0.05, Fig. 1*F*). *PND21* fasting blood glucose was significantly elevated in daughters of Lard OID and Flaxseed OID-fed dams (60 mg/dL and 30 mg/dL higher than control, respectively, Fig. 1*G*). A bolus of sucrose solution was administered via intraperitoneal injection and blood glucose was monitored at 15-, 30-, 60-, and 120-min post-injection (Fig. 1*H*). Female offspring born to dams consuming lard OID had an average area under the curve of 10,000 mg/dL × min higher than female offspring of control-fed animals (*P* < 0.05, Fig. 1*I*).

### In Utero and Early-Life Macronutrient Exposures Initiate Long-Term Impact on the Metabolic Health of Female Offspring

**Figure 2.**
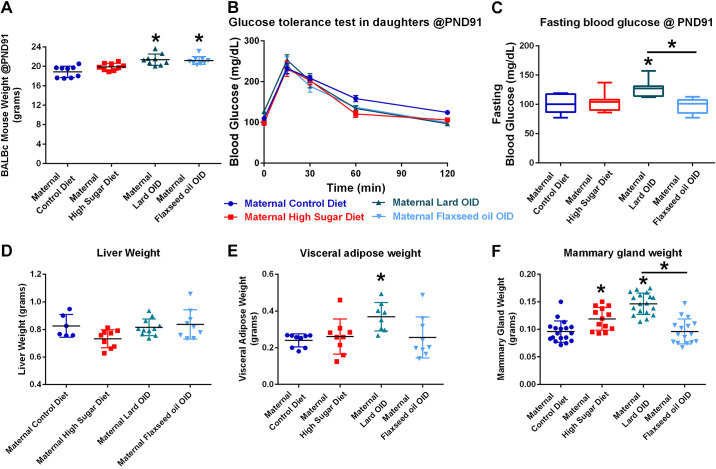
In utero and early-life macronutrient exposures have long-term impacts on the metabolic health of female offspring. *A*: body weights of female BALB/c offspring at 91 days of age. *B*: blood glucose concentration of *postnatal day 91* (*PND91*)-day old female offspring over 2 h following intraperitoneal injection of 2 mg/kg sucrose solution. *C*: comparison of glucose tolerance test curves by calculation of the area under each curve seen in *B*. *D*: wet weight of murine liver tissue at necropsy. *E*: VAT weight of female BALB/c mice at necropsy. *F*: wet weight of BALB/c L4/5 mammary glands at necropsy. *P < 0.05. OID, obesity-inducing diet.

After 10 wk of low-fat control diet exposures (at *PND91*), female offspring born to Lard OID and Flaxseed OID-fed dams had an average of 3 and 2 g higher body weight than offspring of control diet-fed mice, respectively (*P* < 0.05, Fig. 2*A*). At this time, the glucose tolerance testing procedure was repeated and blood glucose curves did not vary between groups (Fig. 2*B*). Mice born to Lard OID-fed mothers had a 1.25-fold elevated fasting blood glucose compared with both daughters of control-fed and Flaxseed OID-fed mice (*P* < 0.05, Fig. 2*C*). No significant differences in liver weight were observed (Fig. 2*D*). Adult offspring of dams fed Lard OID had a 1.5-fold higher VAT weight compared with daughters of control diet-fed mice (*P* < 0.05, Fig. 2*E*). Mammary glands from offspring of HS and Lard OID-fed dams weighed 1.3-fold and 1.5-fold higher, respectively, than those isolated from control-fed dams (*P* < 0.05, Fig. 2*F*). The mammary gland weight of daughters of Lard OID-fed dams also significantly differed from those of Flaxseed OID-fed dams, where Lard OID-fed dams had an average 1.5-fold higher mammary gland weight (*P* < 0.05).

### In Utero and Early-Life Macronutrient Exposures Impact Long-Term VAT Morphology, Fibrosis, and Macrophage Populations in Female Offspring

**Figure 3.**
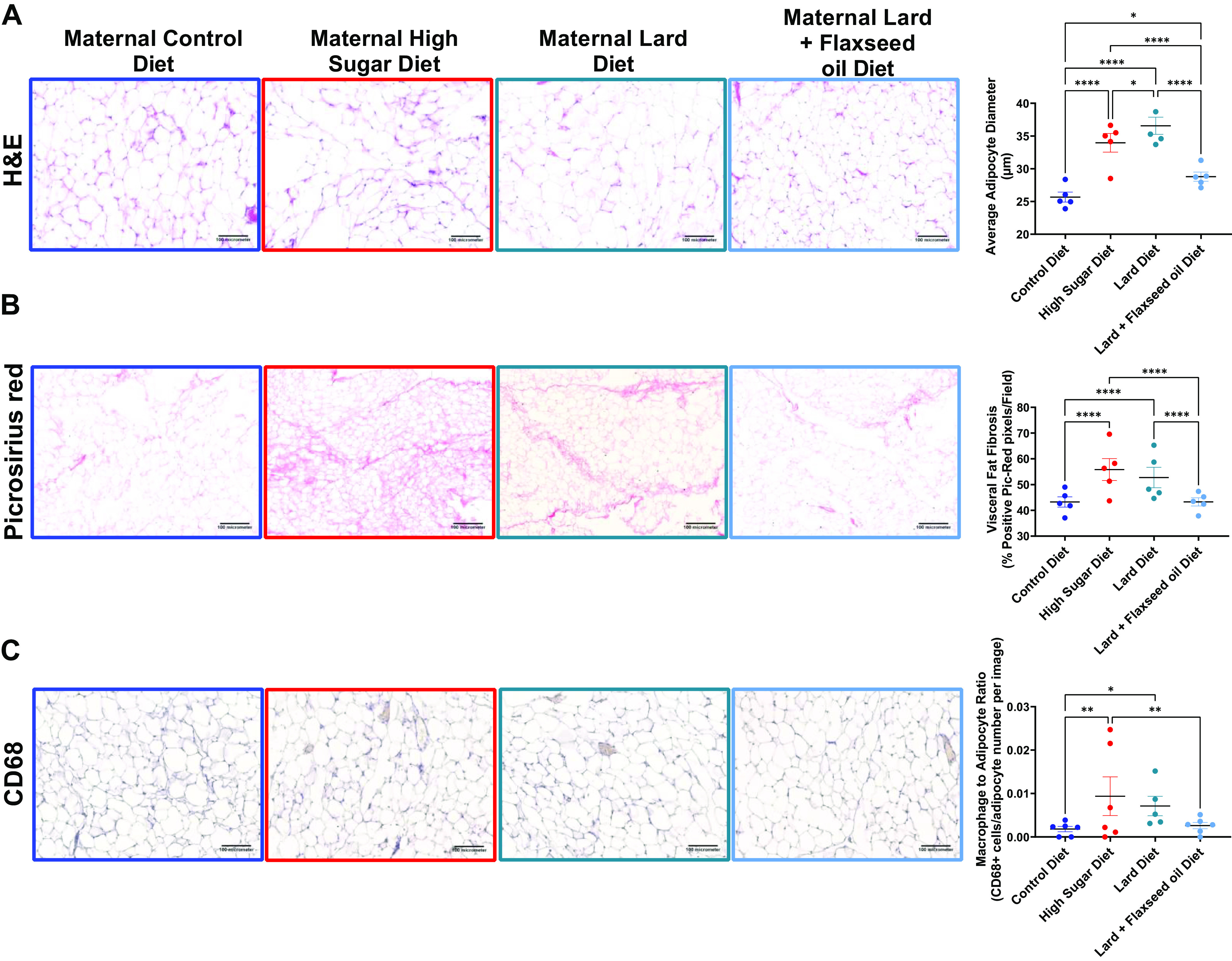
In utero and early-life macronutrient exposures impact long-term VAT morphology and macrophage populations in female offspring. *A*: hematoxylin and eosin staining of VAT harvested from adult female offspring following euthanasia. Adipocyte diameter measurements consist of 20 representative adipocytes in each of five images taken per mouse and *n* = 5 mice. *B*: Picrosirius red (PicRed) staining of collagen in VAT of adult female offspring. Quantification of fibrosis indicates the percentage of pixels that are positive for PicRed staining in five images per animal of *n* = 5 animals. *C*: hematoxylin-DAB staining for CD68 (a pan-macrophage marker) immunoreactivity. Macrophages were counted manually per image for a total of five images each of *n* = 5 or 6 animals. *P < 0.05; **P < 0.01; ****P < 0.0001 averages of groups were compared using two-way ANOVA (main column effect) with a Holm–Šídák’s multiple-comparisons post hoc test. Error bars indicate SEM and the line indicates the mean. H&E, hematoxylin and eosin; VAT, visceral adipose tissue.

Hematoxylin and eosin staining was performed on VAT, and adipocyte diameter was measured (Fig. 3*A*). Maternal consumption of HS, Lard OID, and Flaxseed OID was associated with 1.2-, 1.3-, and 1.1-fold adipocyte hypertrophy compared with the consumption of daughters of control diet-fed mice (*P* < 0.05). However, mice exposed to Lard OID had a 1.1-fold higher adipocyte diameter compared with those exposed to Flaxseed OID (*P* < 0.05). Sections of the VAT were stained with the pan-collagen stain, Picrosirius red, to compare adipose tissue fibrosis (Fig. 3*B*). Exposure to in utero and early-life HS diet was associated with a fourfold elevated VAT collagen deposition compared with daughters of control diet-fed mice (*P* < 0.05). In addition, the offspring of Lard OID-fed mothers exhibited 2.5-fold more VAT collagen content compared with offspring born to Flaxseed OID-fed mothers (*P* < 0.05). IHC staining for the pan-macrophage marker CD68 was performed on VAT sections (Fig. 3*C*). Maternal consumption of HS or lard OID diet but not a Flaxseed OID diet mediated an approximate threefold increase in the VAT macrophage population normalized to adipocyte number.

### Maternal Macronutrient Consumption Modulates Long-Term Gut Microbial Proportional Abundance of Hippurate-Associated Bacteria in Female Offspring

16S sequencing of fecal DNA samples from pregnant BALB/c mice consuming either control, HS, Lard OID, or Flaxseed OID diets at pregnancy *day 10* and from female offspring at *PND21* and *PND91* was performed; principal coordinate analysis (PCoA) demonstrate bacterial community shifts over time and with maternal diet exposures (Supplemental Fig. S1, *A*–*C*), there were no significant differences in Shannon α-diversity between subjects and timepoint (data not shown), and the proportional abundance of identified bacterial phyla were reported (Supplemental Fig. S2*A*). At the phylum level, decreased Bacteroidetes and elevated Firmicutes proportional abundance were observed at weaning (*PND21*) in the daughters from Lard OID-fed dams. However, by adulthood, after a 10-wk administration of the control diet, the phylum level changes were no longer observed (Supplemental Fig. S2, *B* and *C*).

**Figure 4.**
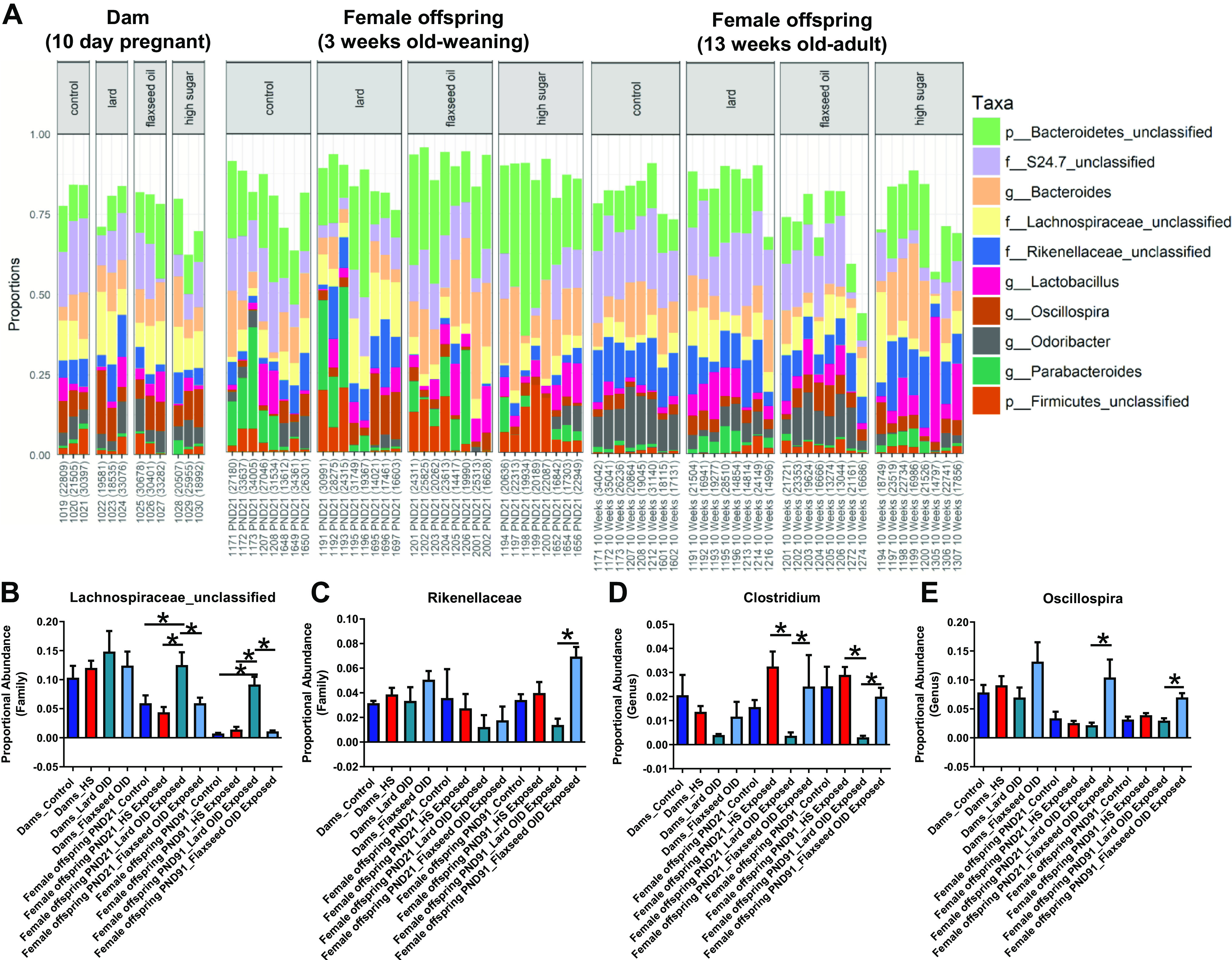
Maternal macronutrient consumption modulates long-term gut microbial levels of hippurate-associated bacteria in female offspring. *A*: proportional abundance of bacterial genera quantified via 16S sequencing in pregnant dams, female offspring at weaning, and adult female offspring (*n* = 3 dams, *n* = 8 daughters). *B*: proportional abundance of *Lachnospiraceae (f)_unclassified* bacteria that are negatively associated with hippurate concentrations are elevated in female offspring from Lard OID-exposed mothers identified in fecal samples by 16S sequencing. Proportional abundance of fecal bacteria that are positively associated with hippurate concentration [*C*: *Rikenellaceae* (family); *D*: *Clostridium*; *E*: *Oscillospira*] are elevated in female offspring from Flaxseed OID-fed dams. *P < 0.05; averages of groups were compared using one-way ANOVA with an uncorrected Fisher’s LSD post hoc test. Error bars indicate SEM. OID, obesity-inducing diet.

Although broad-spectrum shifts to the phyla were not maintained, we did observe significant shifts to several gut microbiota populations on the family and genus level that persisted to adulthood even after control diet administration (Fig. 4*A*). Female offspring from mothers consuming a lard OID displayed twofold elevated *Lachnospiraceae_unclassified* at *PND21* that increased to nine fold at *PND91* compared with controls (*P* < 0.05, Fig. 4*B*). Female offspring from Flaxseed OID-fed dams displayed fourfold increased *Rikenellaceae* (family) compared with offspring from Lard OID-fed dams at adulthood (*PND91*) that was not observed at *PND21* (*P* < 0.05, Fig. 4*C*). Female offspring of Flaxseed OID-consuming mothers also displayed sevenfold elevated *Clostridium* (Fig. 4*D*) and *Oscillospira* (Fig. 4*E*) at weaning (*PND21*) that persisted to 10- and 3.5-fold increases at adulthood (*PND91*), respectively, compared with female offspring of Lard OID-fed mothers (*P* < 0.05).

**Figure 5.**
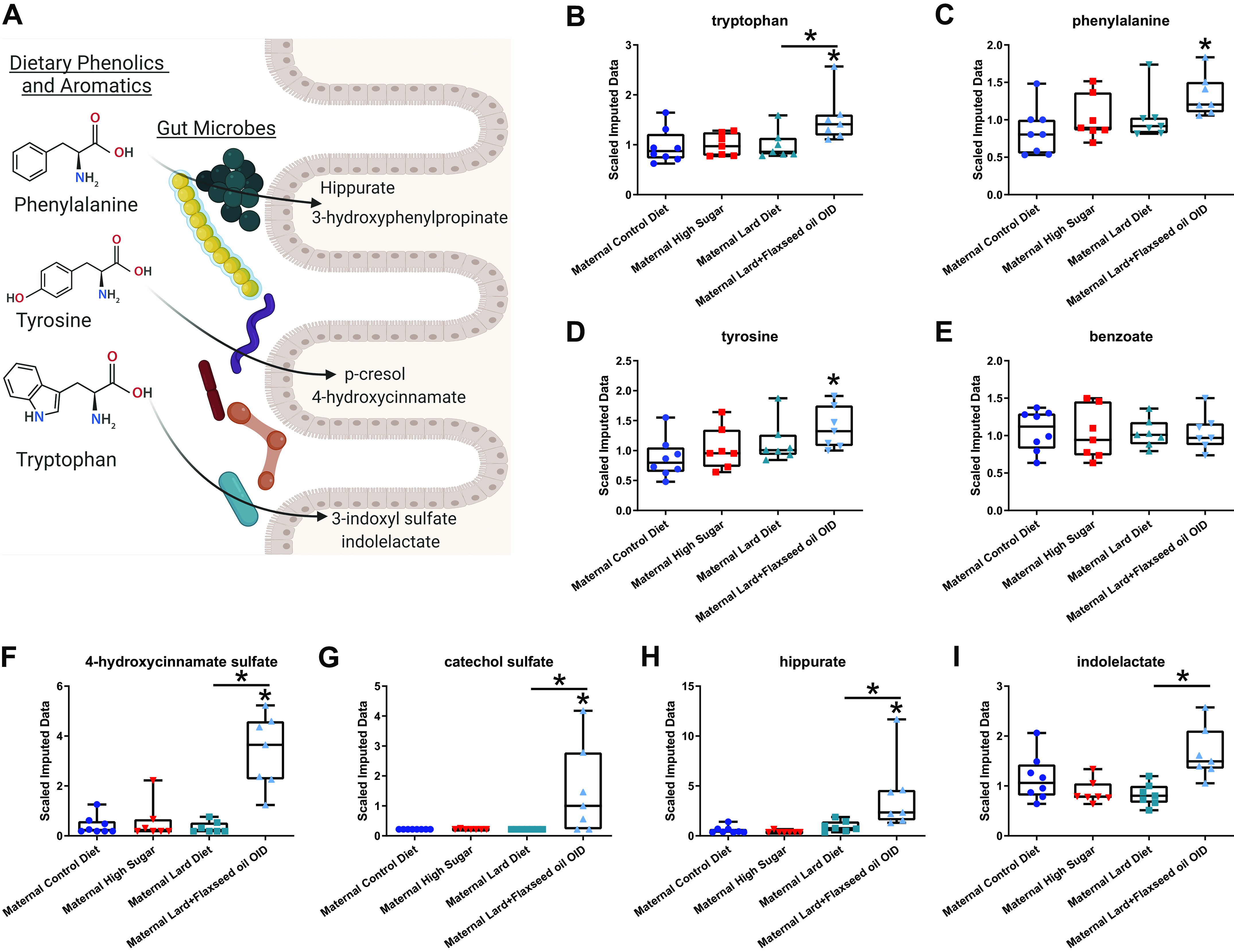
Maternal macronutrient consumption impacts VAT microbial-modified metabolites adult BALB/c female offspring. *A*: dietary phenolic and aromatic amino acids can be converted to form bioactive compounds through a combination of microbiota and host metabolic processes (simplified schematic created using BioRender.com). Scaled data collected via untargeted metabolomics detected parental amino acids [tryptophan (*B*), phenylalanine (*C*), and tyrosine (*D*), and benzoate (*E*)]. Microbial-associated metabolic products such as 4-hydroxycinnamate sulfate (*F*), catechol sulfate (*G*), hippurate (*H*), and indolelactate (*I*) are all elevated in VAT of adult daughters from Flaxseed OID-fed dams. *P < 0.05. *n* = 7 or 8. Averages of groups were compared using one-way ANOVA with a Tukey’s multiple-comparisons test. Error bars indicate SEM. OID, obesity-inducing diet; VAT, visceral adipose tissue.

Previous studies using germ-free and antibiotic-treated mice demonstrated a microbial-mediated metabolite signature where bacteria presence was necessary for the formation of certain metabolites, including hippurate (22–26). Untargeted metabolomics was performed on VAT samples collected from adult female offspring (Fig. 5). In utero and early-life exposure to Flaxseed OID was associated with enriched levels of 4-hydroxycinnamate sulfate (35-fold), catechol sulfate (10-fold), hippurate (10-fold), and indolelactate (1.7-fold) compared with female offspring exposed to Lard OID (*P* < 0.05). With the exception of indolelactate, all metabolites were also significantly enriched in offspring of Flaxseed OID-fed mice compared with control diet-fed mice.

### Hippurate Supplementation Modulates Fibrosis in the VAT of BALB/c Mice

**Figure 6.**
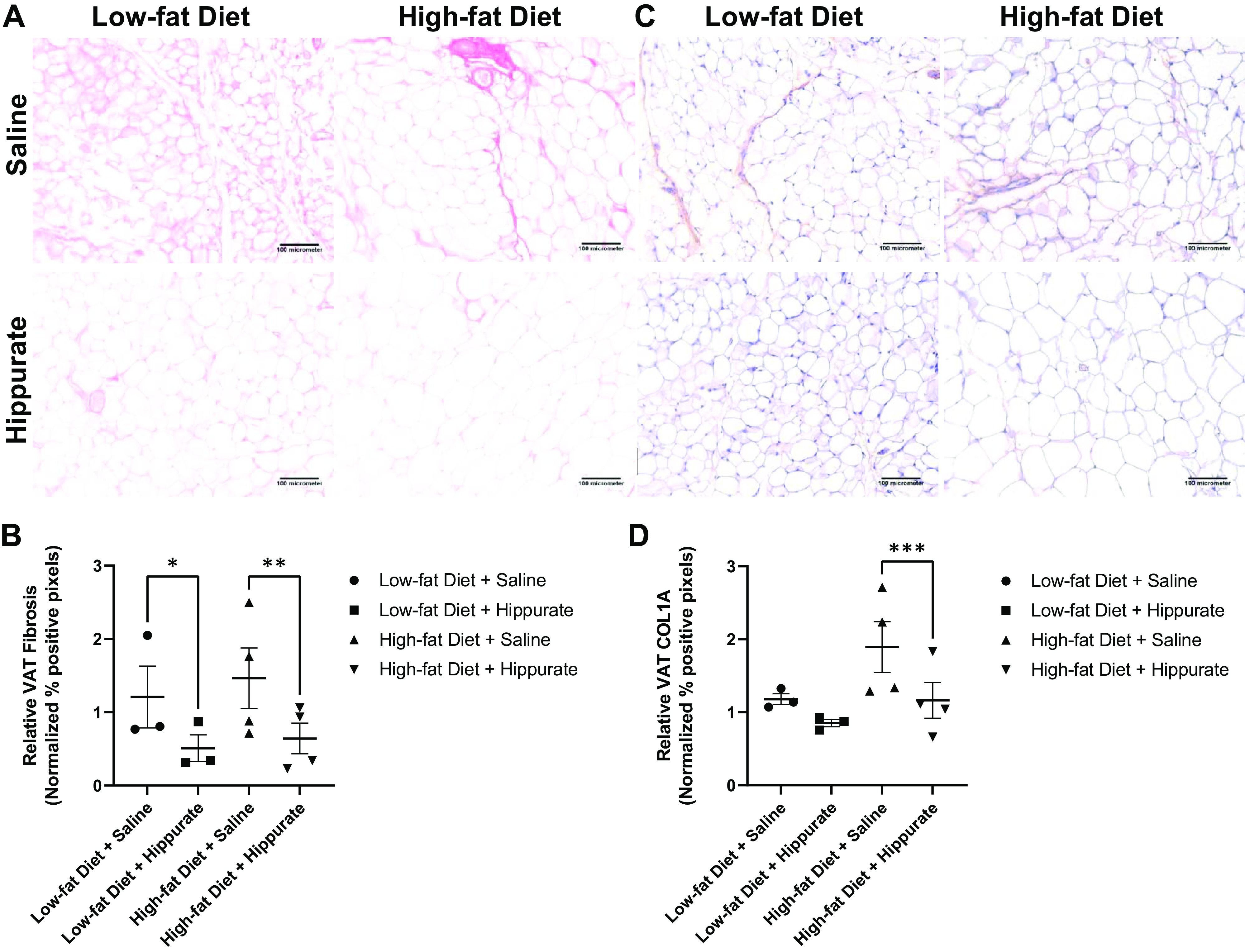
In vivo assessment of microbial-mediated metabolite hippurate on visceral adipose tissue (VAT) pathology. Low-fat or high-fat diet-fed female BALB/c mice were given 3 × 1-wk intraperitoneal injections of 100 µL saline or 10 mM hippurate. Visceral adipose tissue was fixed and embedded in paraffin for physiological examination. VAT was stained using Picrosirius red protocol to visualize fibrosis. *A*: representative images of Picrosirius stained VAT from saline and hippurate-treated low-fat and high-fat diet-fed animals. *B*: quantification of Picrosirius red-stained VAT from saline and hippurate-treated low-fat and high-fat diet-fed animals. Quantification of fibrosis indicates the percentage of pixels that are positive for PicRed staining in five images per animal of *n* = 3 or 4 animals. *C*: representative images of collagen 1A (COL1A)-stained VAT from saline and hippurate-treated low-fat and high-fat diet-fed animals. *D*: quantification of collagen 1A-stained VAT from saline and hippurate-treated low-fat and high-fat diet-fed animals. Quantification of COL1A indicates the percentage of pixels that are DAB chromogen positive in five images per animal of *n* = 3 or 4 animals. *P < 0.05; **P < 0.01; ***P < 0.001; averages of groups were compared using two-way ANOVA (main column effect) with a Holm–Šídák’s multiple-comparisons post hoc test. Error bars indicate SEM.

Female 12-wk-old BALB/c mice were fed a low-fat control diet (*n* = 6) or a high-fat diet (*n* = 8) for 2 wk before being randomized into groups receiving either 10 mM hippurate or a saline placebo (*n* = 3 or 4). Supplementation with exogenous hippurate was associated with an approximate 0.3-fold change in VAT fibrosis as determined by Picrosirius red pixel positivity in both low-fat and high-fat diet-consuming mice (Fig. 6, *A* and *B*). More specifically, VAT sections were stained against collagen 1A (COL1A). Supplementation with exogenous hippurate significantly reduced VAT COL1A deposition in high-fat diet consuming-mice (∼0.5-fold change), whereas there was only a modest trend for reduced COL1A in VAT of low-fat diet-fed subjects (Fig. 6, *C* and *D*). In vivo administration of hippurate in low-fat diet-fed animals decreased the mature TGF-β1 homodimer (25 kDa) (0.5-fold change) and COL1A (0.3-fold change) in VAT as determined by protein densitometry (*P* < 0.05, Supplemental Fig. S3, *A***–***C*).

Ex vivo samples of VAT excised from low-fat diet-consuming mice were either untreated, treated with 10 µM hippurate, or treated with 100 µM hippurate (Supplemental Fig. S3*D*). High-dose exposure to hippurate (100 µM) was associated with reduced pSmad2 (0.3-fold change), TGF-β (0.3-fold change), and COL1A (0.45-fold change) compared with untreated tissue (*P* < 0.05, Supplemental Fig. S3, *E***–***G*). In addition, low-dose hippurate exposure (10 µM) modulated pSmad2 (0.3-fold) and COL1A (0.45-fold) (*P* < 0.05, Supplemental Fig. S3, *E* and *G*).

## DISCUSSION

Early-life programming in response to maternal diet exposure has been implicated as a risk factor for several diseases including neuroendocrine conditions, hypertension, and insulin resistance (27, 28). In previous studies, lard-based maternal high-fat diet (HFD) influenced male rat offspring metabolic health through programming of liver fatty acid metabolism, whereas prenatal exposure to low-fat diet (LFD) protectively programmed the hepatic epigenome to reduce the effects of offspring HFD consumption (29, 30). Maternal HFD’s effects on male offspring liver metabolism have also been observed in murine models where maternal diet influenced postweaning liver metabolite levels (31). In addition, maternal flaxseed oil consumption during lactation enhanced bone development in male rat pups (32). Although most of the previous studies investigated male offspring as a model of maternal diet, some studies identified sexually dimorphic responses to prenatal exposure, indicating the need for further research investigating the response to maternal diet in female offspring. For instance, although both male and female murine offspring developed an elevated body fat percentage in response to maternal HFD, only female offspring had reduced oxidative phosphorylation capabilities (33). In addition, an investigation using a Wistar rat model reported that maternal OID consumption is associated with accelerated offspring metabolic aging in all offspring but is differentially regulated in males and females (34). The maternal consumption of flaxseed had sexually dimorphic effects in rat offspring, including differences in glucose metabolism, pancreatic cellular morphology, and aortic remodeling (35–38). In a study using SM/J mice exposed to maternal HFD or LFD and then weaned onto either HFD or LFD, female HFD-fed offspring of HFD-fed mothers had an exacerbated response to their diets and quickly gained more weight and adipose tissue than other groups (39). In our study, we provide a novel contribution to the field by examining the impact of in utero and early-life macronutrient exposure on gut microbial populations and their associated metabolites as a potential mechanism for maternal diet-associated adverse metabolic conditions in female offspring.

In the Healthy Start study, a poor diet during pregnancy was correlated with increased neonatal adiposity in humans regardless of maternal BMI before pregnancy (40). We report that exposure to Lard and Flaxseed OID was associated with an elevated birth weight of mouse pups, echoing these results. Studies using C57BL/6 mice demonstrated that the offspring of obese murine dams are often heavier than those of nonobese dams, even up to 6 mo of age (27). Our study uses a BALB/c model, which is known to be an obesity-resistant model. However, we were able to replicate the long-term impacts of in utero and early-life exposure to a Lard OID and Flaxseed OID on body weight in this model. Maternal diet-induced obesity in C57BL/6 mice correlated with insulin resistance of offspring in a model using a lard-based OID with supplementation of sweetened condensed milk (27). An examination of monosodium glutamate (MSG)-induced obesity, primarily in male mice, demonstrated that prenatal flaxseed exposure through maternal diet blocked glucose intolerance, insulin resistance, pancreatic islet dysfunction, and elevated fat content associated with obesity (41). Similarly, we report impaired glucose metabolism in female offspring of BALB/c mice exposed to maternal Lard OID. Our results indicate that alteration of the n-6:n-3 polyunsaturated fatty acid (PUFA) ratio by addition of flaxseed oil to a Lard OID (Flaxseed OID) is protective against this effect. This result corroborates an existing study in which female offspring of HFD and streptozocin-induced diabetic rat dams fed a high flaxseed oil diet had reduced fasting blood glucose compared with daughters of diabetic mothers consuming a control diet or HFD (38).

A study using a C57BL/6 mouse model reported increased adiposity in adult offspring associated with maternal consumption of a lard-based OID (27). In addition, results in murine models reported inflamed adipose tissue in male offspring in response to maternal macronutrient consumption that was reduced by intervention with an LFD during pregnancy (42). Our study expands this knowledge at the tissue level by characterizing the adipose tissue and reporting altered hypertrophy, fibrosis, and macrophage content in response to in utero and early-life macronutrient exposures. Of these effects, both fibrosis and hypertrophy correlate with obesity and increased the risk of metabolic disease, specifically diabetes, in humans with obesity (43, 44).

A study of male and female Sprague–Dawley rat pups of obese dams treated with a prebiotic supplement indicated that control of the maternal gut microbiota can influence metabolic factors in both mothers and offspring (45). Prenatal exposure to the oligofructose prebiotic ameliorated glucose tolerance, insulin sensitivity, and hepatic steatosis in offspring of dams consuming a high fat/sucrose diet (46). At weaning, offspring were offered a high-fat/sucrose diet, and gut microbial composition was examined. The study identified initial maternal diet-associated mediation of gut bacterial taxa (*Akkermansia muciniphila, Bifidobacterium, Enterobacteriaceae*, and *Lactobacillus*) that were not observed in fecal DNA at 11 wk of age, whereas maternal diet had a long-term impact on gut colonization of *Clostridium leptum* and *Roseburia*. This study supports our observation that maternal diet impacts gut microbiome shifts that can persist long term that are associated with metabolic outcomes. Weight gain in the offspring of murine dams fed a lard-based HFD was previously associated with gut microbial taxa (*Lachnospiraceae, Clostridiaceae, Aldercreutzia, Coprococcus*, and *Lactococcus*) and male offspring specifically had significantly enriched gut *Firmicutes* that are positively correlated with obesity risk (47). Studies of direct flaxseed oil consumption in rats have indicated favorable changes in gut microbial populations including a reduced *Firmicutes/Bacteroidetes* ratio (48, 49). However, maternal dietary consumption of flaxseed oil has not previously been linked to gut microbial populations of adult offspring. We further examined the influence of maternal diet on offspring gut microbial populations and indicate a novel effect of in utero and early-life exposure to Lard and Flaxseed OID by reporting their effects on the gut microbial contents of female offspring. Hippurate is a glycine conjugate derived from the metabolism of polyphenols to benzoate by the gut microbiome (50–53). We identified specific regulation of hippurate-associated bacterial taxa and report that Lard OID positively correlates with fecal *Lachnospiraceae*, which were previously negatively correlated with hippurate concentration and positively associated with weight gain in offspring of HFD-fed dams (47, 54). We show that elevated fecal *Lachnospiraceae* in daughters of Lard OID-fed dams had decreased hippurate concentrations in the VAT supporting this previous association. In addition, we indicate that supplementation of flaxseed oil into a Lard OID (Flaxseed OID) is in turn positively associated with bacterial taxa (*Rikenellaceae, Oscillospira*, and *Clostridium*) that were seen to be positively correlated with VAT hippurate concentration. Of these taxa, *Rickenellaceae* has been positively correlated with hippurate concentration in human samples (54). High levels of hippurate were reported to be favorable; hippurate is associated with high fruit and grain intake and is positively correlated with gut microbial diversity (54). Elevated circulating hippurate is associated with the expression of neuroglobin by the adipose tissue, which has been shown to protect cells from hypoxia and oxidative stress in other tissues (55, 56), Low hippurate levels have also been reported to be unfavorable in inflammatory and microbial-associated conditions, as both Crohn’s disease and obesity are reported to be negatively correlated with hippurate concentration (57–60). We hypothesized that this effect may indicate that hippurate is the metabolite responsible for the protective effects of Flaxseed OID versus Lard OID that we observed and examined this using in vivo and ex vivo models.

In a murine model of HFD-fed mice receiving chronic subcutaneous hippurate infusion, hippurate was associated with improved glycemic control, improved insulin secretion, reduced liver inflammation, and reduced liver fibrosis (61). We report that hippurate supplementation reduces the expression of TGF-β and COL1A in VAT. TGF-β is associated with immune cell activity through its functions in wound-healing and fibrosis by helping to initiate the chemotaxis of macrophages and fibroblasts to a body site (62, 63). Reduction of TGF-β in response to hippurate is a potential mechanism by which Flaxseed OID may reduce VAT fibrosis and macrophage infiltration in our model. Similarly, the reduced collagen observed in the adipose tissue of hippurate-treated BALB/c mice is correlated with the reduced fibrosis observed in the VAT of mice receiving a Flaxseed OID compared with Lard OID. VAT fibrosis is common in chronic obesity, where fibrotic tissue can accumulate in response to chronic inflammation; it is characterized by reduced VAT plasticity and poor angiogenesis, leading to further dysregulation of insulin signaling (43). Ex vivo analysis confirmed the results observed in our hippurate treatment model and revealed a reduction in pSmad2 mediated by hippurate exposure. Smad family proteins participate in TGF-β signal transduction pathways and are similarly correlated with inflammation and fibrotic responses when activated by phosphorylation (64–66). Direct consumption of flaxseed oil by rats was previously shown to have protective effects against pulmonary fibrosis, whereas maternal diet exposure to a flaxseed oil diet correlated with reduced kidney fibrosis (67, 68). However, our study is the first to report an impact of maternal flaxseed oil exposure on VAT fibrosis.

In conclusion, our study indicates that in utero and early-life macronutrient exposure yields long-term effects on the metabolic and gut microbial health of female offspring. We also report that decreasing the n-6:n-3 PUFA ratio by supplementation of a lard-based high-fat diet with flaxseed oil (Flaxseed OID) is protective against some of the observed unfavorable effects. We propose microbial effects as a mechanism for this, specifically an increase in bacteria positively correlated with hippurate. Finally, we report that hippurate supplementation can reduce VAT fibrosis, suggesting exogenous administration of hippurate may be efficacious at reducing adverse health effects associated with obesity.

Our future studies will examine the mechanisms related to the effects observed with early-life exposure to a high sugar diet using our model (adipose fibrosis, adipocyte diameter, mammary gland weight). We will also further examine the mammary glands collected from these animals. A previous study in female Sprague–Dawley rats reported that a maternal western-style diet enhanced the effects of chemically induced mammary tumors through changes to the transcriptome (69). In a C57BL/6J mouse model of 7,12-Dimethylbenz[a]anthracene-induced mammary tumors in daughters prenatally exposed to flaxseed, safflower, or fish oil diets, the daughters of mothers consuming n-3 PUFA (flaxseed oil and fish oil) had reduced breast cancer risk compared with those consuming safflower oil (70). Our results also indicate that maternal macronutrient consumption may predispose female offspring to develop breast cancer risk factors. Elevated body weight and adipose tissue inflammation are risk factors for breast cancer (71). In addition, both TGF-β and Smad2 are indicators of poor prognosis in human breast cancers (72–75). For these reasons, we propose examining the impact of maternal macronutrient consumption on female offspring breast cancer risk as our forthcoming focus for this project.

A potential limitation of our study is the use of only female offspring. Sex differences are observed in the development of obesity, and what we demonstrate in our female cohorts may be differentially regulated in male cohorts. Another important point to discuss is the distinction between gestational and lactational exposures. In the current study, we have grouped subjects by maternal diet as early-life exposures since this encompasses both in utero and lactational exposures. Breast milk has been shown to contain microbiota and microbial metabolites that can influence gut colonization of offspring. Therefore, determining gestational versus lactational differences is key for future studies using a dam cross-fostering approach with different dietary exposures. Finally, the associations between gut microbiome species and VAT hippurate concentrations are largely correlative. Future experimentation involving antibiotic administration and fecal microbiota transplants is needed to definitively demonstrate causality between the gut microbiome and hippurate in our preclinical models.

## DATA AVAILABILITY

All data generated or analyzed during the described study are included in this published article and supplemental materials.

## SUPPLEMENTAL DATA

10.6084/m9.figshare.20204036 Supplemental Fig. S1: https://doi.org/10.6084/m9.figshare.20204036.

10.6084/m9.figshare.20204564 Supplemental Fig. S2: https://doi.org/10.6084/m9.figshare.20204564.

10.6084/m9.figshare.20204819 Supplemental Fig. S3: https://doi.org/10.6084/m9.figshare.20204819.

## GRANTS

This work was supported by the American Cancer Society Research Scholar Grants (RSG-16-204-01-NEC to K.L.C. and 133727-RSG-19-150-01-LIB to D.R.S.-P.), a grant from the Susan G. Komen Foundation (CCR18547795 to K.L.C.), National Institutes of Health National Cancer Institute (NIH NCI) (R21CA249349 to D.R.S.-P.), American Society for Radiation Oncology-Breast Cancer Research Foundation (ASTRO-BCRF) Career Development Award (637969 to D.R.S.-P.), and Breakthrough Award from the Department of Defense Breast Cancer Research Program (W81XWH-20-1-0014 to K.L.C.). K.Y.J.C. is a recipient of a T32CA247819 training award. Shared Resource services were provided by the Wake Forest Baptist Comprehensive Cancer Center’s NCI Cancer Center Support Grant P30CA012197.

## DISCLOSURES

No conflicts of interest, financial or otherwise, are declared by the authors.

## AUTHOR CONTRIBUTIONS

K.L.C. conceived and designed research; T.M.N., K.Y.J.C., and A.S.W. performed experiments; T.M.N., K.Y.J.C., A.S.W., and K.L.C. analyzed data; A.S.W., D.R.S.-P., H.M.O.-B., and K.L.C. interpreted results of experiments; T.M.N., K.Y.J.C., and K.L.C. prepared figures; T.M.N. and D.R.S.-P. drafted manuscript; D.R.S.-P., H.M.O.-B., and K.L.C. edited and revised manuscript; T.M.N., K.Y.J.C., A.S.W., D.R.S.-P., H.M.O.-B., and K.L.C. approved final version of manuscript.
